# Design and evaluation of blended teaching in the smart classroom combined with virtual simulation training in basic nursing courses

**DOI:** 10.1186/s12909-023-04721-3

**Published:** 2023-10-11

**Authors:** Ya Meng, Jian Song, Xiaojing Yu, Xiaoxia Xu, Hao Zhang

**Affiliations:** 1 School of Medical, Huanghe Science and Technology College, Zhengzhou, 450000 China; 2 School of Nursing, Jingzhou Vocational and Technical College, Jingzhou, 434020 China; 3 College of Education, Soochow University, Suzhou, 215127 China; 4 The Affiliated Cancer Hospital of Zhengzhou University, Zhengzhou, 450008 China

**Keywords:** Smart classroom, Virtual simulation training, Blended teaching design, Independent learning ability

## Abstract

**Objective:**

This study explored the application effect of smart classrooms combined with virtual simulation training in basic nursing courses for nursing undergraduates.

**Methods:**

In this quasi-experimental study, a total of 135 undergraduate nursing students in the 2021 matriculating cohort were selected as the research subjects. The experimental group of Class 1 had 71 students, and a blended teaching design utilizing a smart classroom and virtual simulation training was adopted. The control group of Class 2 had 64 students, and traditional lecture-based teaching design was adopted. After the course, the independent learning ability scale, test scores and teaching effectiveness questionnaire were used to evaluate the teaching effect. All tests had a maximum score of 100.

**Results:**

Nursing undergraduates in the experimental group had scores of 86.32 ± 8.25 for virtual simulation training and 84.82 ± 9.04 for peer-assisted learning. The scores of the theoretical examination, experimental examination, and subjective questions in the experimental group were significantly higher than those in the control group (*P* < 0.05). The approval rate of nursing undergraduates in the experimental group was significantly higher than that of the control group for four items (*Ps* < 0.05). Among the 71 students, most students (91.55%) claimed that the use of instructional designs increased the fun of the classroom. In addition to the dimension of information literacy, the total score of independent learning ability and the other three dimensions of the experimental group were significantly higher than those of the control group (*P* < 0.05).

**Conclusion:**

The teaching design combining smart classrooms and virtual simulation training can be applied to realize online blended teaching and classroom informatization, improving the academic performance and independent learning ability of nursing undergraduates, and thus achieving good teaching effects.

## Background

In December 2021, China’s “14th Five-Year Plan” for national informatization proposed the construction of an integrated internet + education cloud network to build a ubiquitous online learning space. In the same year, the 5G application “sailing” action plan called for increasing the application of 5G in smart classrooms and combining augmented reality/virtual reality (AR/VR), holographic projection and other technologies to realize scene-based interactive teaching and create immersive classrooms [1, 2]. A smart classroom is characterized by natural human–computer interaction and is an enhanced classroom realized by technologies such as mobile communication, cloud computing, big data and more [3]. Smart classrooms, as an important part of the process of implementing intelligent teaching, are characterized by abundant resources, extensive interaction, a combination of virtual and real-life environments, and rich functions, thus opening up a new field for the creation of teaching situations [4]. Such classrooms rely on the panoramic data recording and analysis of the teaching process in the form of a “cloud + terminal”, the visual presentation of and feedback on results, and the provision of digital teaching tools and the establishment of a decision-making basis for teaching and learning.

With the development and construction of smart classrooms, research on blended teaching has emerged. The research on online and offline hybrid teaching models conducted by foreign scholars mainly focuses on classroom technology implementation, teaching model construction, and teaching service provision [5]; that conducted by domestic scholars, however, is mainly aimed at the construction of new teaching models and the enrichment of teaching methods [6–8]. In seeking to improve students’ enthusiasm for learning by combining flipped classrooms, massive open online courses (MOOCs), and microclasses, teachers are now using teaching cloud platforms to monitor students’ learning performance, observe students’ learning needs and habits, adjust their teaching methods, and deliver personalized lessons [9, 10].

It is well known that the traditional classroom teaching mode is mainly aimed at imparting knowledge, which is characteristically teacher-centered and emphasizes the teacher’s dominant position and leading role, making it an insufficient method of recording students’ learning process and cultivating their cooperation and communication skills. Compared with the advantages of traditional classrooms, those of smart classrooms are mainly reflected in the following aspects. First, the configuration of information hardware and software is very complete, the teaching environment is better, and the learning atmosphere is more relaxed and novel. Second, the learning platform can help teachers manage their courses and engage in real-time interactions through random roll calls and classroom tests. Third, a smart classroom focuses on generating student knowledge so that the presentation and pedagogical approach to content are more diversified, encouraging students to engage in adaptive learning and participate in cooperative group teaching [9, 11]. At present, the widely used smart teaching tools, such as rain classrooms, micro teaching assistants app, and super star learning app, are only the application of mobile phone software in ordinary classrooms or online courses and rarely involve smart classrooms. [12, 13].

Additionally, VR is receiving increasing attention in nursing education and is being used to teach many nursing concepts, including leadership, communication, decision-making, critical thinking, inclusion, health assessment, and disaster classification [14]. Using virtual simulation technology to build a virtual operating environment and provide students with multisensory simulation [15], real-time interactions will help solve difficulties in implementing experimental projects, such as high costs, high levels of risk, and difficult operations. In addition, researchers have incorporated case-based learning (CBL) and peer-assisted learning(PAL) into the teaching design process [16]. In CBL, teachers create learning situations with typical cases, and students read cases and collect data to learn through scenario simulation, carry out discussion and analysis, and synthesize knowledge related to various cases to cultivate clinical thinking ability. While PAL is a kind of learning mode in which peers support and assist each other, which plays a positive role in improving students’ academic performance and developing social emotions [17].

Basic nursing courses are some of the most fundamental and important courses in nursing programs. They are introductory courses encompassing professional clinical knowledge and are a cornerstone of nursing education and teaching. Therefore, in the teaching process of basic nursing courses, constructing a hybrid teaching model based on smart classrooms and exploring a new mechanism for the integration of science, education and collaborative learning are necessary and effective ways to improve teaching quality.

In summary, based on CBL assisted by smart classroom technology and virtual simulation training, this study aims to enrich the mixed teaching method and help teachers flexibly inform their teaching in the hope of improving students’ ability to discover, analyze and solve problems.

## Methods

A quasi-experimental study design was adopted, including an experimental and control group with baseline and follow-up assessment.

### Participants

G-power was used to verify the sample size. The *t* test was selected under the test family (effect size convention = 0.5, 1-β error probability = 0.95, α error probability = 0.05), and the total sample size was 54. Finally, a total of 135 nursing undergraduates from the 2021 cohort of the Huanghe Science and Technology College were selected as the research objects through cluster sampling. There were 71 students in the experimental group in the first class and 64 in the control group in the second class. The age of the experimental group members ranged from 19 to 23 (20.80 ± 0.86) years old, and the age of the control group members ranged from 19 to 23 (20.63 ± 0.93) years old; the difference in age distribution was not statistically significant (*t* = 1.154, *P* = 0.250). The experimental group consisted of 60 women and 11 men, and the control group consisted of 60 women and 4 men; the difference was not statistically significant (*χ*^2^ = 2.912, *P* = 0.088). The two groups of students had already studied pharmacology, physiology, biochemistry, anatomy, and other professional basic courses in their program.

### Teaching methods

Traditional teaching methods were used by the control group. Theoretical teaching is mainly based on teacher lectures, while experimental teaching is carried out through the teaching process of watching instructional videos, teaching, and helping students practice.

### Teaching design for the experimental group

This study involved combining theoretical teaching with virtual simulation training and PAL; engaging in online and offline hybrid teaching; analyzing and designing the learning content, process, activities, and evaluation of the experimental group; and promoting the comprehensive improvement of nursing undergraduates. The specific operation process was as follows:

### Teaching preparation

Teacher preparation: (1) Teachers were tasked with optimizing classroom equipment (e.g., interactive electronic whiteboards, multiple interactive screens replace projection screens), replacing fixed desks and chairs with movable combined desks and chairs, using interactive touch-screen electronic blackboards, and improving and upgrading voice collection and other automation equipment to help achieve full coverage of Wi-Fi high-density nodes, solve network congestion, prevent students from becoming disconnected, and enable the transmission of classroom resources and feedback learning status in real time [18]. (2) Microclasses such as oral care, aseptic techniques, occupational protection, catheterization, the handling of gowns, intravenous infusion, enemas, catheterization, and cardiopulmonary resuscitation were recorded to assist classroom teaching; clinical cases for oral care, hospital infection prevention and control, aseptic techniques, intramuscular injection, intravenous infusion and other key teaching points and content difficulties were noted. (3) Online classes and WeChat groups for smart teaching tools were created. According to the curriculum standards and curriculum teaching plan, learning task sheets were issued one week before the theoretical class, and teaching videos, clinical cases, teaching materials, microclasses, preclass test questions, and reference books were uploaded. Teachers were also instructed to answer student questions online, monitor the progress and results of preclass learning, and adjust the classroom teaching design in a timely manner. (4) Teachers cooperated with information technology companies to select virtual simulation training items such as units on enemas, catheterization, and cardiopulmonary resuscitation and to import information such as student names and student numbers into the virtual simulation training system. (5) A scoring sheet for PAL was created and a corresponding QR code for each item was created through the PAL app, which was used for assessment during experimental operation exercises.

Student preparation: (1) Students first had to become familiar with the course design process of blended teaching, the navigation and use of the online learning platform app, the precautions for virtual simulation training, course evaluation instructions, etc. They scanned the QR code to join the class and submit their personal information. (2) Online discussions were conducted. Students watched specific microclass videos, conducted online discussions to complete preclass tests and exercises, summarized difficult problems, and posted them on the class discussion board [19]. (3) Students were asked to complete group tasks. Based on situational teaching, students were divided into groups of 5–6 people and assigned roles according to the results of case discussions. Then, they shot situational simulation videos or prepared PPTs for case analysis of theoretical classroom reports.

### Teaching materials

#### Teaching implementation

##### Theory teaching

The teacher opened the smart classroom and the students scanned the QR code to sign into and access the classroom. The teacher then summarized and reviewed common problems in preparation for the preclass test and previewed them through discussion. Then, according to the teaching content and the corresponding clinical cases presented in the chapter, the teacher asked exploratory questions to prime the learning atmosphere. Students played scenario simulation videos or made case analysis reports through group PPTs, and teachers and other students then shared comments and discussed the content for approximately 5 min.

Based on traditional teaching, other teaching activities applied in the classroom included online quizzes in the Rain Classroom. That is, teachers published practice experimental group for the relevant chapter in a timely manner according to the most important and difficult points that would come up on the test to dynamically understand what the students had learned. Test question types included multiple-choice questions, voting questions, subjective questions and more, and the students’ aggregate answers could also be shown on mobile phones and multimedia screens in real time.

In addition, teachers could interact with students through the Rain Classroom platform to stimulate the learning atmosphere. For example, teachers could assess a student’s mastery of the lesson by randomly calling on students, and students could post short responses about a given issue. Teachers rewarded students who answered questions quickly and correctly by giving them red envelopes. Notably, the Rain Classroom platform used informatization means to record real-time and comprehensive teaching process data on sign-ins, interactions, performance levels, and correct answers to questions for nursing undergraduates, which was a very convenient way for teachers to obtain statistics on the teaching process in later stages.

##### Virtual simulation training

After the theoretical class was over, nursing undergraduates moved to the virtual simulation laboratory for nursing, logged in to the virtual simulation training system using their student number, and practiced the training items corresponding to the content of the theoretical class. Training, proficiency in operating procedures and precautions were all covered topics.

##### Practice in groups for experimental items

Once students had moved to the nursing practice room, they formed random pairs. One student used a tablet or mobile phone to scan the QR code through the PAL app to obtain the electronic score sheet for the project. Combined with the process and scoring standards of the score sheet, the first student then supervised the other student, ensured the standardization of the operation, corrected incorrect methods or steps in the operation, and evaluated the effect of the exercise, and the software recorded and exported the training results.

##### Summary feedback

The teacher summarized the key points and difficulties in the class, evaluated the students’ classroom performance and problems, and helped students master knowledge more deeply and systematically.

**Fig. 1:**
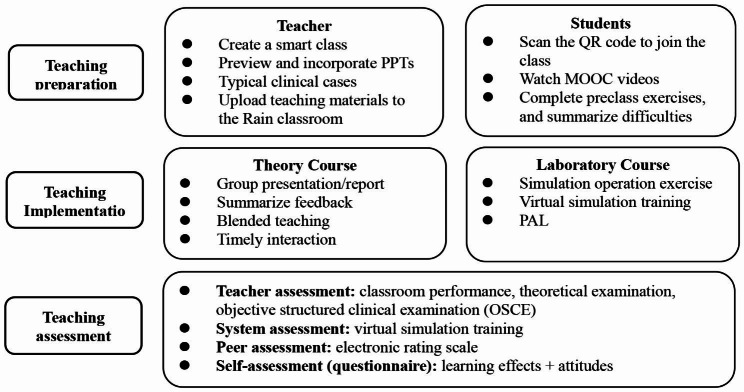
Teaching design chart

The implementation process is shown in Fig. 1.

#### Instruments

##### Academic performance

The grades for each unit were scored using the percentile system. The results of virtual simulation training and PAL are finally calculated on a percentage basis.

##### Theoretical assessment

The content of the theoretical assessment in the experimental group and the control group were the same, and a closed-book examination was held at the end of the semester with a total score of 100. The types of questions were divided into objective questions (40 single-choice questions scored at 1 point each and 10 multiple-choice questions scored at 2 points each), and there were two types of subjective questions (5 short-answer questions scored at 4 points each and 2 case analysis questions scored at 10 points each).

##### Experimental assessment

The control group drew lots to determine the test items, which took the form of short case studies. After the operation was implemented, the teacher gave an objective score. The experimental group took an Objective Structured Clinical Examination (OSCE) [20]. Four test stations were set up, and each station was equipped with an electronic case system and a number calling system. Candidates tested one skill at each station, namely, cardiopulmonary resuscitation, intravenous infusion, intramuscular injection and catheterization, within 10 min. The teacher set up numerous assessment cases, and the undergraduate nursing undergraduates swiped their ID cards for inspection and then waited in the waiting room for the system to randomly call their numbers. After confirming the test items, they entered different OSCE stations. Nursing evaluation, planning and health education were assessed through students’ oral reports, and nursing measures were assessed in the form of scenario simulations. Nursing measures were the focus of the experimental assessment. Teachers used tablet computers and the electronic scoring form corresponding to the assessment cases to record notes and provide scores for the students. The test at each station had a maximum score of 100, and the average score of the four stations was used as the experimental assessment score.

#### Questionnaire survey

After the course teaching was implemented, a questionnaire developed by the researchers was used to evaluate the effects of instructors’ teaching. The questionnaire was completed through the “wenjuanxing” platform and uploaded to the learning platform in the form of a QR code. Students scanned the code to answer the questions after confirming their informed consent. The content of the questionnaire included the following: (1) General information, including age, gender, place of origin, etc., was collected. (2) Nursing undergraduates’ independent learning ability was evaluated using a scale compiled by Zhang Xiyan [21] in 2009 that included learning motivation (8 items), self-management ability (11 items), learning cooperation ability (5 items), and information quality (6 items). The scale had 4 dimensions, 10 secondary structural elements, and a total of 30 items, and responses were given on a 5-point Likert scale ranging from 1 (“completely inconsistent”) to 5 (“completely consistent”). Reverse statement items were reverse scored, and the total scores ranged from 30 to 150 points. The higher the score was, the stronger the independent learning ability. (3) A questionnaire was used to investigate the teaching effects (8 items) and attitudes (6 items) of nursing undergraduates, and there were three response options for each item: “agree,” “neutral” and “disagree”. The evaluation of the smart classroom by nursing undergraduates in the experimental group was assessed using 6 items.

### Statistical analysis

The original survey data were exported from the “Wenjuanxing” platform and combined with students’ academic performance and virtual simulation training results to form the final database. SPSS 26.0 software was used for the statistical analysis of the data. The descriptive analysis of measurement data was expressed as the mean ± standard deviation (x ± s), and the comparison between groups was performed by *t* test and analysis of variance. The descriptive analysis of count data was described in terms of rate (%), and the between-group analysis was conducted using the *χ*^*2*^ test with a significance level of 0.05 (two-sided).

## Results

### Academic performance of nursing undergraduates in the experimental group

The experimental group consisting of seventy-one nursing undergraduates had total scores of 61–98 (86.32 ± 8.25) in virtual simulation training and 63–97 (84.82 ± 9.04) in peer-assisted learning.

### Comparison of theoretical assessment and experimental assessment between the two groups

The scores for the theoretical assessment, experimental assessment, and subjective questions in the experimental group were significantly higher than those in the control group (*P* < 0.05). There was no significant difference between the two groups in the scores for the objective questions (*P* > 0.05) (Table 1).

**Table 1:** Comparison of scores between the two groups ($\bar x$± s, points)

Item	Experimental group (n = 71)	Control group (n = 64)	*t* value	*P* value
Score of theoretical assessment	82.54 ± 8.08	77.58 ± 8.65	3.429	0.001^*^
Objective questions	48.00 ± 6.42	47.33 ± 7.31	0.568	0.571
Subjective questions	34.54 ± 3.87	32.02 ± 4.44	3.524	0.001^*^
Score of experimental assessment	85.61 ± 7.85	81.83 ± 10.84	2.297	0.023^*^

^*^There was a significant difference between the two groups (*P* < 0.05)

### Comparison of scores of independent learning ability

After implementing the teaching design that combines the smart classroom with virtual simulation training, the total score of independent learning ability and the dimensions of learning motivation, ability to learn and collaborate, and self-management skills of the experimental group, as well as the level of their information literacy, were significantly higher than those of the control group (*P* < 0.05) (Table 2).

**Table 2:** Comparison of the scores for independent learning ability between the two groups ($\bar x$± s, points)

**Groups**	**Learning motivation**	**Self-management skills**	**Ability to learn and collaborate**	**Information literacy**	**Total score**
Experimental group (n = 71)	31.65 ± 3.39	42.41 ± 6.20	19.79 ± 3.03	22.03 ± 3.28	115.87 ± 11.71
Control group (n = 64)	30.06 ± 4.45	40.05 ± 5.03	18.25 ± 2.82	21.22 ± 4.38	109.58 ± 10.16
*t* value	2.341	2.414	3.044	1.224	3.319
*P* value	0.021^*^	0.017^*^	0.003^*^	0.223	0.001^*^

^*^There was a significant difference between the two groups (*P* < 0.05)

### Evaluation of the teaching effects

In the evaluation of the teaching effects for the experimental group, the highest percentage of agreement was for improving independent learning ability (88.7%), and the lowest was for promoting the cultivation of professional attitudes (63.4%). In the evaluation of the traditional classroom teaching effects of the control group, 79.7% of the nursing undergraduates stated that they had conscientiously worked through the learning content, but only 43.3% of the nursing undergraduates reported a belief that traditional classrooms create an active classroom atmosphere.

In terms of teaching effects, both groups agreed that the teacher had effectively guided and managed the class and that the students had conscientiously worked through the learning content. There was no significant difference between the groups (*P* > 0.05). The approval rate of nursing undergraduates in the experimental group was significantly higher than that of the control group for the four items, including “Stimulate the enthusiasm and initiative of learning” (*Ps* < 0.05) (Table 3).

**Table 3:** Comparison of teaching effects between the two groups (n = 135)

Item	Experimental group (n = 71)				Control group (n = 64)			Fisher’s exact probability χ^2^	*P* value
	Agree n(%)	Neutraln(%)	Disagreen(%)		Agree n(%)	Neutral n(%)	Disagreen(%)		
Stimulate enthusiasm and initiative for learning	56(78.9)	13(18.3)	2(2.8)		39(60.9)	15(23.4)	10(15.6)	8.148	0.016^*^
Cultivate a professional attitude	45(63.4)	16(22.5)	10(14.1)		34(53.1)	21(32.8)	9(14.1)	1.914	0.394
Improve analytical and problem-solving skills	55(77.5)	10(14.1)	6(8.5)		41(64.1)	8(12.5)	15(23.4)	5.725	0.059
Enhance knowledge and understanding and improve learning efficiency	61(85.9)	9(12.7)	1(1.4)		37(46.5)	8(8.1)	19(29.7)	23.895	< 0.001
Improve independent learning ability	63(88.7)	6(8.5)	2(2.8)		42(65.6)	11(17.2)	11(17.2)	11.561	0.003^*^
Create an active classroom atmosphere	59(83.1)	8(11.3)	8(11.3)		35(43.3)	12(9.2)	17(11.5)	9.268	0.009^*^
The teacher has effectively guided and managed the class.	53(74.6)	11(15.5)	7(9.9)		42(65.6)	12(18.8)	10(15.6)	1.504	0.470
I have conscientiously worked through the learning content.	60(84.5)	7(9.9)	4(5.6)		51(79.7)	8(12.5)	5(7.8)	0.621	0.793

^*^There was a significant difference between the two groups (*P* < 0.05)

### The attitude of nursing undergraduates in the experimental group

**Fig. 2:**
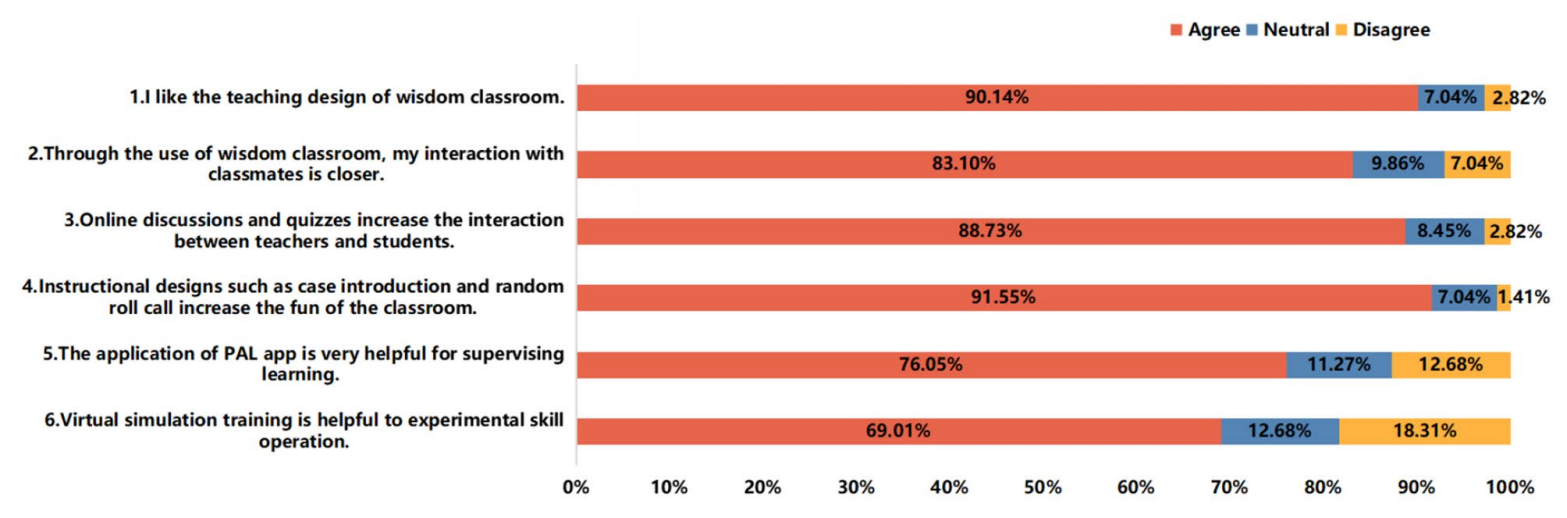
Evaluation of experimental group nursing undergraduates regarding their obtained knowledge class (n = 71)

Figure 2 summarizes the experimental group nursing undergraduates’ evaluation of the effect of the teaching method of smart classrooms combined with virtual simulation training. Among the 71 students, 64 (90.14%) liked the teaching design of smart classrooms.

In addition, 83.10% and 88.73% of the students, respectively, believed that the smart classroom improved interactions with their classmates and that online discussions and quizzes on the Rain Classroom platform increased the number of interactions between teachers and students. Moreover, most students (91.55%) noted that instructional designs such as live subtitles, random roll calls, and red envelopes increased the fun of the classroom. However, 18.31% and 12.68% of the students thought that virtual simulation training was not helpful for experimenting with skills and that the application of the PAL app was not very helpful for supervising learning, respectively.

## Discussion

Smart classrooms represent a new type of classroom produced by the deep integration of information technology and traditional classroom teaching [22]. These classrooms assist with sharing teaching resources, optimizing the collection and sharing of teaching materials, recording the teaching process, and organizing and reporting student grades. Teaching evaluations are produced through a comprehensive dynamic evaluation of the entire teaching process. The hybrid teaching mode based on the smart classroom has been very convenient for both teachers and students, helping undergraduate nursing students choose the times and places that are most convenient for them to learn with the support of modern information technology and form personalized learning strategies that increase their own initiative in the learning process [23].

In addition, it is worth noting that smart classrooms can challenge teachers’ ability to apply information technology. Teachers need to fully understand their students’ learning progress, motivate and guide them, and teach them in accordance with their own needs and abilities. This requires teachers to comprehensively and deeply understand how to use information technology, such as proficiency with virtual simulation training systems and PAL app, particularly the Rain Classroom, which is used for sharing preclass information, producing online coursework, integrating MOOCs and online videos, providing real-time feedback in class, facilitating teacher-student interaction, and providing postclass summaries [24]. The Rain Classroom integrates complex information technology into PowerPoint and WeChat and builds a communication bridge between preclass content previews and classroom teaching [25, 26].

At the same time, to adapt to ever-changing information technology developments, teachers should continually update their teaching concepts and methods, actively obtain the latest knowledge and skills, and pay attention to new developments in the nursing industry at home and abroad, which is also of great significance in comprehensively building a body of teaching staff. In summary, only teachers with strong learning ability, a strong sense of responsibility, patience and the courage to try new technologies and new things can be successful in teaching under this new model [25].

### Teaching effect

The results of this study show that the theoretical test scores and experimental scores of the test group are significantly higher than those of the control group, suggesting that the hybrid teaching mode, which combines smart classroom and virtual simulation training, is helpful for improving the theoretical and practical knowledge of nursing undergraduates. This is vital for the following reasons. First, in blended teaching, undergraduate nursing students are no longer seen as pure knowledge recipients but rather as active learners who can communicate with their teachers and classmates at any time through the online learning platform. Instantaneous communication supports the personalized learning needs of students and helps them to learn, understand and deeply master knowledge. Second, theoretical classes, case analysis and group scenario simulation reports can help to improve the preclass and in-class achievements of group members with low grades and enhance students’ overall academic performance [27]. Teachers can follow up on important and difficult problems using Rain Classroom tests, and students’ answers can be directly projected on the screen in the form of a histogram, helping students to strengthen their knowledge retention and teachers to quickly grasp what students have learned and to adjust their teaching priorities accordingly. Third, in experimental classes, virtual simulation training and practical operation training are combined, and simulated scenarios are used to conduct nursing diagnosis as well as health education, nurse‒patient communication, standard operating procedures and preventative actions to strengthen the level of understanding and mastery of students [28]. Fourth, online learning resources, such as microvideos and virtual simulation training, can be reviewed repeatedly using corresponding test questions for each topic that facilitate the review and consolidation of important and difficult knowledge for nursing students [29, 30].

### Independent learning ability

With the rapid development of medical technology, the cultivation of nursing undergraduates’ autonomous learning ability has far-reaching significance for the future of nurses’ lifelong learning. The survey results showed that 88.7% of the nursing students in the experimental group believed that the new teaching method could improve their independent learning ability; this figure was significantly higher than that in the control group (*P* < 0.05).

Tao et al. defined the independent learning ability of nursing undergraduates as the ability to use metacognition to acquire and master the knowledge and skills necessary to provide high-quality nursing services [31]. Previous studies have shown that improving the environment and equipment that is used in independent learning can improve teaching designs and subsequently improve students’ capacity for independent learning [32–34]. The smart classroom adopts teaching designs such as preclass preview, collaborative group case analysis and scenario simulations, which require students to actively acquire and internalize knowledge by consulting materials, analyzing and discussing information, and developing good learning habits. At the same time, it is worth noting that throughout the learning process, nursing undergraduates constantly use mobile phones and network resources to access and process information online [35, 36], thereby greatly improving the quality of consulted information. The convenience of mobile phones and the internet assist in the asynchronous and nonlocalized learning of nursing students, who no longer solely rely on classroom learning, a move that can effectively improve students’ capacity for autonomous learning [37]. In addition, the establishment of process evaluation indicators such as scores for PAL and virtual simulation training can further cultivate that capacity.

### Teaching satisfaction

Compared with traditional teaching (43.3%), 83.1% of nursing undergraduates in the experimental group believed that smart classrooms can create an active classroom atmosphere, which is very useful for stimulating enthusiasm and initiative in learning. A total of 69.01% of nursing undergraduates in the experiment thought that virtual simulation training was helpful in improving their operational skills. Virtual simulation training to simulate the clinical work situation can provide more practice opportunities at a low cost and make abstract theoretical knowledge more intuitive and vivid [38]. Game-like operation modes also increase learning fun, more deeply immerse students, and increase levels of student satisfaction. An active classroom atmosphere helps to improve students’ concentration and expressiveness, assisting them in the transition from passive to active learning [39, 40].

More than 80% of the nursing students in the experimental group believed that the use of smart classrooms made the interactions among students closer, especially through the mechanisms of short posts, random roll calls, online discussions and quizzes, which increased not only the levels of communication and interaction between teachers and students but also the level of fun in the classroom, thereby significantly improving the activity level in the classroom and the level of student participation.

### Limitations

Some limitations should be noted.

First, smart classrooms have high requirements in terms of classroom facilities and network speeds. If the requirements cannot be met, many teaching designs cannot be implemented. Given the excellent teaching effect of smart classrooms, it is suggested that more schools should be equipped with smart teaching tools.

Second, smart classrooms combined with virtual simulation training place a higher burden on teachers. Our suggestions to overcome this are as follows: First, an efficient and cooperative teaching team should be established, the task distribution and division of labor should be clarified, and high-quality resources should be integrated. At the same time, the teaching team should cooperate with information technology companies to improve the digital function of the platform and thereby reduce the burden on teachers.

Third, this study was conducted at only a single university in Henan Province. Therefore, it did not have a large sample size or cover different regions. Thus, it is suggested that a large-scale study be conducted to verify the merits of the proposed teaching design through more robust empirical evidence.

## Conclusion

The deep integration of information technology and teaching and the establishment of a blended teaching model combining smart classrooms and virtual simulation training can improve the academic performance, independent learning ability, learning effect, and satisfaction of nursing undergraduates as well as teaching efficiency and teaching quality, making it worthy of application and promotion.

## Data Availability

All data generated or analyzed during this study are included in this published article.
